# Functional Fibers, Composites and Textiles Utilizing Photothermal and Joule Heating

**DOI:** 10.3390/polym12010189

**Published:** 2020-01-10

**Authors:** Juhyun Park

**Affiliations:** School of Chemical Engineering and Materials Science, Institute of Energy-Converting Soft Materials, Chung-Ang University, Seoul 06974, Korea; jpark@cau.ac.kr

**Keywords:** photothermal conversion, Joule heating, reduced tungsten oxide, tungsten bronzes, conjugated polymer nanomaterials, silver nanowires, smart textiles

## Abstract

This review focuses on the mechanism of adjusting the thermal environment surrounding the human body via textiles. Recently highlighted technologies for thermal management are based on the photothermal conversion principle and Joule heating for wearable electronics. Recent innovations in this technology are described, with a focus on reports in the last three years and are categorized into three subjects: (1) thermal management technologies of a passive type using light irradiation of the outside environment (photothermal heating), (2) those of an active type employing external electrical circuits (Joule heating), and (3) biomimetic structures. Fibers and textiles from the design of fibers and textiles perspective are also discussed with suggestions for future directions to maximize thermal storage and to minimize heat loss.

## 1. Introduction

Textiles that have specific functions added to their inherent properties have been some of the most interesting themes for academic and industrial studies and development [1,2,3]. Functional textiles originate from attempts to improve the properties of textiles based on synthetic fibers and to make their properties similar to textiles based on natural fibers. For example, synthetic fibers generally have lower hydrophilicity than natural fibers, such that clothes made from synthetic fibers generally retard the removal of sweat and moisture from the human body to the surrounding environment. Various chemical and physical methodologies, including hydrophilic surface modifications [4,5,6], blending with hydrophilic fibers [7,8], co-polymerizations with hydrophilic monomers [9,10,11], and increasing fiber surface area by introducing porosity [12,13,14,15] or capillaries [16,17,18,19], have been applied to synthetic fibers to enhance the absorption and removal of moisture and water. These treatments significantly enhance the soft and pleasant feeling of wearing clothes fabricated with such modified synthetic fibers. These days, the scope of application of functional textiles has been further extended to clothes for health care [20,21,22], medical treatments [23,24,25], sports and leisure [26,27,28], environmental pollution [29,30,31], and wearable optoelectronics [32,33,34,35]. Therefore, special functions including antimicrobial [36,37,38,39], warming or cooling [40,41,42], waterproof [43,44,45], or windproof properties [46,47] need to be incorporated into the fibers. As a representative technology, photocatalytic nanomaterials such as titanium dioxide nanoparticles have been combined with fibers to degrade odor-causing chemicals and microbial species under light irradiation to remove any unhealthy odor or bacteria [48,49,50]. In addition, textiles that have not been wet by rain or snow but can transport sweat across clothes, have been fabricated by laminating, coating, or densely packing fibers to provide micropores or hydrophilicity, thereby providing waterproof and windproof properties and maintaining body temperature by controlling the transport of heat, water, and moisture [40,41,42,43,44,45,46,47].

Among the many functionalities for textiles, the particular focus of this review is the mechanism of adjusting the thermal environment surrounding the human body via textiles. A traditional method for coping with hot or cold weather is to take off or put on clothes. Numerous state-of-art technologies, however, have enabled the development of functional textiles to maintain warmth or to dissipate heat for cooling. Commercial products with these functionalities are already available in the market. One of the most popular products is “Heattech” developed by Uniqlo Co., Ltd. and Toray Industries, Inc. in 2003 [51]. Heattech uses textiles based on acrylic fibers with a circular cross-sectional shape and rayon fibers with a sharply edged cross-sectional shape. Because both fibers are hydrophilic, the resulting textiles efficiently absorb and maintain water molecules from sweat, thereby hindering heat loss by water evaporation into air and rather releasing heat upon adsorption of water molecules onto hydrophilic fibers. At the same time, the mismatch in the cross-sectional shapes between the acrylic and rayon fibers results in increased amounts of air pockets in yarns, resulting in a decrease in heat transfer across the textiles because of the low thermal conductivity of the air. Other types of popular products utilize radiation from the human body. The “Omni-Heat” developed by Columbia Sportswear Co. uses fibers coated with silver or aluminum, which reflect heat from the human body while efficiently releasing moisture and sweat across the textile structure, thereby providing warmth and comfort [52]. In contrast, phase change materials (PCMs) have been widely studied and applied to commercial products because of their controllability over heat absorption and emission. PCMs with a melting transition are embedded in fibers as microcapsules [53,54], and thus, as an example, the materials experience heat absorption due to melting transition upon temperature increase and heat emission due to crystallization upon temperature decrease in the surrounding environment. 

Recently, novel technologies based on the photothermal conversion principle and Joule heating for wearable electronics have been highlighted as promising technologies for future functional fibers and textiles. They should be an important element for personal thermal management by raising the human body’s temperature when applied to normal clothes. Furthermore, heating fabrics and composite films have become promising materials in biomedical and cosmetic applications. This is because thermotherapy can open blood vessels and increase blood flow, bringing nutrients for healing wounds, as demonstrated by a thermoelectric heat patch for clinical care based on Joule heating [55]. In addition, they can significantly accelerate the transdermal deliveries of medicines or cosmetic agents when put in contact with human skin by providing fluidity to the epidermis at an elevated temperature, thereby increasing mass transportation across the human skin [56,57,58]. Furthermore, photothermal heating materials such as gold nanorods were loaded in the stratum corneum of the skin and presented photothermally-driven antibacterial activity [59], and a thermoelectric heat patch based on Joule heating. In this review, we focus on the recent innovation in these technologies over the last three years. Such a timely review is necessary due to the numerous papers regarding this issue that have been reported.

This review is organized as follows: Section 2 provides an overview of the methodologies based on photothermal heating, focusing on materials that can efficiently absorb solar energy under sunlight irradiation and emit the energy as heat. These technologies should be useful for the fabrication of clothes for harsh and cold environments where clothes are naturally exposed to sunlight. Inorganic materials such as zirconium-based materials are first described, followed by an introduction of recent studies using metal oxides and conjugated polymers with excellent light harvesting and photothermal properties. Section 3 details the recent innovations in functional fibers and textiles based on the Joule heating principle. The incorporation of electrical conductors with resistance into fibers is a direct method for controlling the thermal properties of textiles based on electrically resistive heating. These technologies are some of the most widely studied, in combination with wearable displays, sensors, and therapeutic devices. Section 4 analyzes fibers and textiles from a viewpoint of the design of fibers and textiles. In Section 5, the recent innovations are summarized, and future directions are suggested. 

## 2. Functional Fibers, Textiles, and Composites Based on Photothermal Heating

### 2.1. Photothermal Heating by Inorganic Materials

Metals, metal oxides, metal carbides, and metal sulfides have been widely used for photothermal solar energy conversion because of their efficient light absorption properties based on surface plasmon resonance in metals [60], sub-band transitions in metal oxides [61,62], and match in inherent frequencies of ceramics for resonance with light [58]. In this section, a few examples of recently reported metal oxides and ceramics are discussed, while metallic materials are described in the section based on the Joule heating principle.

**Figure 1:**
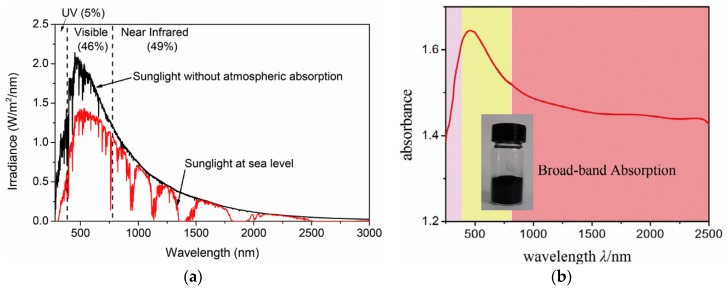
(**a**) Standard terrestrial solar spectral irradiation distributions by the American Society for Testing and Materials (ASTM G173). Reproduced from Ref. [64] with permission. Copyright 2017, Elsevier, and (**b**) absorption spectrum of ZrC nanoparticles. Reproduced from Ref. [67] with permission. Copyright 2017, Elsevier.

Zirconium carbide (ZrC) is one of the most popular ceramics for photothermal applications based on solar energy conversion. It has been widely used as a coating in nuclear reactors, abrasive parts, and cutting tools because of its excellent refractory properties, relatively low density (6.73 g/cm^3^ at 24 °C) compared to that of other carbides, high melting point (~3530 °C), and high modulus (~440 GPa) and hardness (25~35 GPa) [63]. It has been highlighted as an efficient photothermal material because its light absorption spectrum overlaps significantly with the sunlight spectrum that reaches the surface of the earth (Figure 1a,b) [64,65,66,67]. The sunlight that reaches sea level on earth is comprised of 5% ultraviolet (UV), 46% visible, and 49% near infrared (NIR) light [64]. ZrC can absorb a vast amount of solar energy below a wavelength of less than 2 μm and can convert it to heat. Recently, ZrC fluids containing only 0.02 wt % of ZrC nanoparticles were reported to absorb almost 100% of the solar irradiation in the full spectrum, thus showing that ZrC is an ideal solar irradiation absorber [67]. The incorporation of ZrC into polymeric fibers and textiles has been limited because of high processing temperatures, although ZrC-only nanofibers can be conveniently fabricated via a pyrolysis process after electrospinning from precursor polymer solutions. Textiles or fabrics with a ZrC coating, however, can be easily prepared because ZrC films can be deposited via sputtering [68], pulsed-laser deposition [69], chemical vapor deposition [70], or e-beam deposition processes [71]. The resulting polyester fabrics with a ZrC coating are heated to a maximum of 52.5 °C with a temperature increase rate of 11.0 °C/min under infrared lamp illumination [72]. Technologies to incorporate ZrC nanomaterials into polymeric fibers and textiles remain rare and need to be actively developed in the future. 

Reduced tungsten oxides and tungsten bronze nanomaterials are also efficient photothermal materials. The band gap of tungsten trioxide (WO_3_) is 2.62 eV, which makes it suitable for photovoltaic and photocatalytic applications that require strong UV-vis absorption [73], but it is not appropriate for NIR absorption. When WO_3_ is reduced to WO_3−x_, however, a unique defect structure due to oxygen vacancies is introduced with the partial reduction of W^6+^ [74]. In contrast, when alkali metal ions (M = Li^+^, Na^+^, K^+^ and Cs^+^) are incorporated into the crystal structure of WO_3_, a part of W^6+^ in the WO_3_ crystal is reduced to W^5+^, generating sub-bands in its conduction band [75,76]. Both reduced states form localized surface plasmon resonance and sub-band transitions that enable the reduced WO_3_ and M_x_WO_3_ materials to strongly absorb NIR and emit heat [77]. In addition, nanometer-scale reduced WO_3_ materials present a strongly enhanced photothermal conversion property because of the increase in surface area and the resulting surface plasmon resonance [78]. Recently, these reduced WO_3_ nanomaterials have been used for biomedical applications due to their photothermal properties [79,80,81]. Cancer cells are more susceptible to local heating than normal cells. Thus, targeting photothermal materials to cancer tissues, followed by light irradiation to ablate the cancer cells by heat emitted from the photothermal materials, has been a popular area of study as a promising technology for cancer therapy. In addition, photoacoustic imaging that utilizes pulsed laser irradiation and subsequent local density fluctuation upon heating, followed by the generation of acoustic waves, has been an efficient diagnostic tool. Tissues and blood components scatter light strongly, and therefore, the use of NIR should be a solution by providing energy from an outside source to photothermal nanomaterials targeted to cancer tissues inside the human body. This is because NIR can be efficiently transmitted into the human body and is the reason the reduced WO_3_ nanomaterials have been tested for photothermal therapeutic applications. 

**Figure 2:**
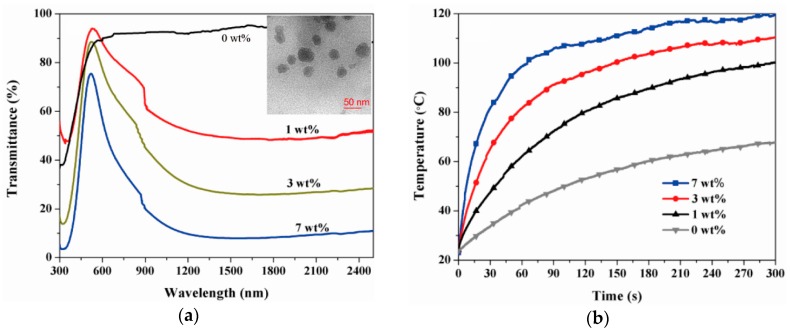
(**a**) UV-vis-NIR transmittance spectra of WO_2.72_/polyurethane (PU) nanocomposites prepared with different weight fractions of WO_2.72_ (0–7 wt %) with an inserted image of cross-sectional high-resolution transmission electron microscopy (HR-TEM) at 7 wt % and (**b**) corresponding photothermal temperature distribution at the different weight factions. Reproduced from Ref. [82].

**Figure 3:**
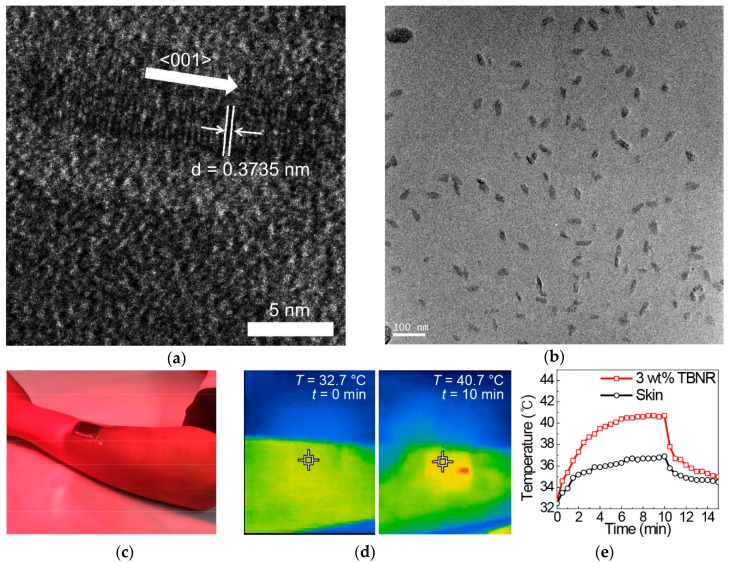
HR-TEM images of (**a**) Na_0.33_WO_3_ TBNRs, (**b**) EPDM/TBNR nanocomposites with 3 wt % TBNR, (**c**) an optical image of an arm with a patch of a nanocomposite film with 3 wt % TBNRs under NIR irradiation by a medical NIR lamp, (**d**) NIR images of the arm at 0 and 10 min under NIR irradiation, and (**e**) photothermal temperature increases of the skin and patch including 3-wt % Na_0.33_WO_3_ TBNRs under NIR light irradiation for 10 min, followed by temperature decreases after turning off the light. Reproduced from Ref. [83].

Applications of these reduced WO_3_ nanomaterials to polymeric fibers and textiles have rarely been observed. Recently, the applications of polymer nanocomposites as solar collectors, functional coatings, and energy-saving applications were reported. Cheng et al. prepared nanocomposites of reduced tungsten oxide and polyurethane (PU) and showed that the photothermal temperature increases, depending on WO_3−x_ concentrations and the degree of reduction [82]. Nanoparticles of reduced WO_3_ with a sub-100 nm diameter were prepared by ball milling WO_3_ powders, followed by their reduction in a tubular furnace under a CO atmosphere. WO_3−x_ nanoparticles were then mixed with PU in dimethyl formamide (DMF), resulting in WO_3−x_/PU nanocomposites upon solvent drying. At a 7 wt % concentration of WO_2.72_, the polymer nanocomposites presented a photothermal temperature increase of up to 120 °C, with an extremely high heating rate of approximately 100 °C/min under IR irradiation at a power of 150 W (Figure 2). Park et al. reported polymer nanocomposite films of tungsten bronze nanorods (TBNRs, Na_0.33_WO_3_) and ethylene propylene diene monomer (EPDM) [83]. TBNRs with a 14-nm length, a 2- to 3-nm width, and an oleyl amine surface capping layer were synthesized via one-pot solvothermal decomposition of ammonium metatungstate hydrate. It is noteworthy that TBNRs were synthesized when sodium ions were introduced into the crystal structure of WO_3_, but only tungsten bronze nanoparticles (TBNPs, Cs_0.33_WO_3_) were prepared when cesium ions were intercalated. Polymer nanocomposites containing both TBNRs and TBNPs at 3 wt % concentration showed a photothermal temperature increase of 3.5–3.7 °C/min, reaching approximately 40 °C in 2.5 min of NIR irradiation from a solar simulator (Figure 3). It was noted that polymer nanocomposites with 3 wt % TBNRs showed a tensile strain of 165%, which was higher than pristine EPDM films which showed a tensile strain of 120%. All other polymer nanocomposites with 1, 2, and 3 wt % of TBNPs and 1 and 2 wt % of TBNRs presented poorer tensile strains than those of the pristine film. Angle-dependent small angle X-ray scattering and HR-TEM experiments revealed that the autophobic dewetting phenomenon due to entropic penalty of short surface alkyls in comparison to long polymer chains in the EPDM matrix caused micrometer-scale aggregation of TBNRs at 1 and 2 wt % concentrations. These aggregates could act as sites for stress concentration upon tensile strain experiments, resulting in mechanical failure at low tensile strains. In comparison, at 3 wt % of the TBNR content, TBNRs formed ellipsoidal particulates with a 46-nm length and 20-nm width that were evenly distributed in the EPDM matrix because of the alleviation of entropic penalty with increasing TBNR concentrations. Reports for fibers, yarns, and textiles fabricated via a melt extrusion or solution spinning process utilizing photothermal inorganic nanomaterials as fillers for polymer nanocomposites are currently limited. The results in the study of TBNR/EPDM nanocomposites suggest that photothermal inorganic nanorods can be suitable for manufacturing fibers and yarns via conventional melting and solution processes due to enhanced mechanical properties at low filler contents. 

### 2.2. Photothermal Heating by Conjugated Polymer Nanomaterials (CPNs)

**Figure 4:**
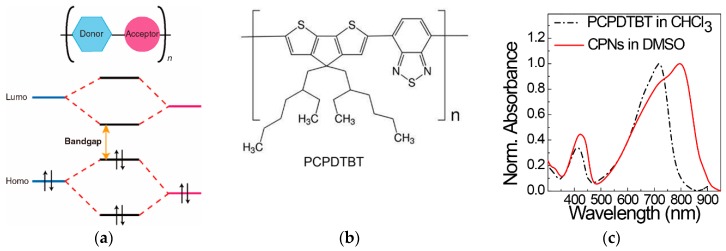
(**a**) Simplified electronic structure of donor-acceptor copolymer chains with a low bandgap. Reproduced from Ref. [95] with permission. Copyright 2017 John Wiley and Sons, (**b**) chemical structure of PCPDTBT and (**c**) its absorption spectra in chloroform solution and CPNs. Reproduced from Ref. [102].

Conjugated polymers that have π-delocalization along their conjugated backbones have been one of the most useful semiconductors for optoelectronic devices such as light-emitting diodes [84], thin-film transistors [85], and photovoltaic cells [86]. In particular, when the structural units of both a donor and an acceptor comprise a repeating unit of the conjugated polymers, intramolecular charge transfer exists from the donor structure unit to the acceptor unit, thereby suppressing charge recombination between electrons and holes and light emission [82,83,84,85,86,87,88]. The subsequent intra- and intermolecular charge transfer processes emit heat rather than light, thus making the donor-acceptor-type conjugated polymers ideal materials for photothermal energy conversion applications [64,87,88,89,90,91,92,93,94]. Furthermore, such donor-acceptor-type conjugated polymers have narrow band gaps, enabling the efficient absorption of NIR (Figure 4a) [95]. In contrast, conjugated polymers can be formed as nanomaterials of nanoparticles and nanoellipsoids in polar solvents of water, low molecular weight alcohols, DMF, and dimethyl sulfoxide (DMSO) [96]. In contrast to conventional thin-film coating processes using solutions of conjugated polymers in good solvents such as toluene, chlorobenzene, and chloroform, nanoprecipitation [97,98], emulsification [98], in-situ polymerization [99], and shattering of phase-separated films to nanomaterials [100] in poor polar solvents could provide CPNs in the polar solvents. Thus, CPNs can be widely examined as photothermal therapeutic agents, photoacoustic probes, and photocatalysts in aqueous-based applications. In addition, they could be incorporated into functional fibers via a solution spinning process utilizing DMF and DMSO [101,102]. Another advantage of CPNs is that they can present optoelectronic properties similar to those of their bulk or thin-film states. When conjugated polymers such as polyelectrolytes are dissolved in polar solvents at a molecular level by incorporating ionic charges into the molecular structures of conjugated polymers, they are directly exposed to the surrounding dipoles, water, and oxygen molecules. Energy is therefore absorbed by the conjugated polyelectrolytes from irradiated light, which typically causes oxidation and degradation of the conjugated backbone, deteriorating their original optoelectronic properties [95]. In comparison, conjugated polymer backbones inside CPNs are isolated from the surrounding polar media, and thus, their optoelectronic properties can be significantly preserved. In addition, the stacking of π-conjugated backbones as in films of conjugated polymers with ordered assembly structures is possible, resulting in a bathochromic shift in their absorption spectra by intermolecular π-electron delocalization, as shown in the absorption spectra of poly[*2*,*6*-(4,4-bis-(2-ethylhexyl)-4*H*-cyclopenta[2,1-b;3,4-b′]-dithiophene)-*alt*-4,7-(2,1,3-benzothiadiazole]] (PCPDTBT) (Figure 4b,c) [102]. As a result, the NIR absorption and heat emission properties of CPNs are significantly enhanced.

**Figure 5:**
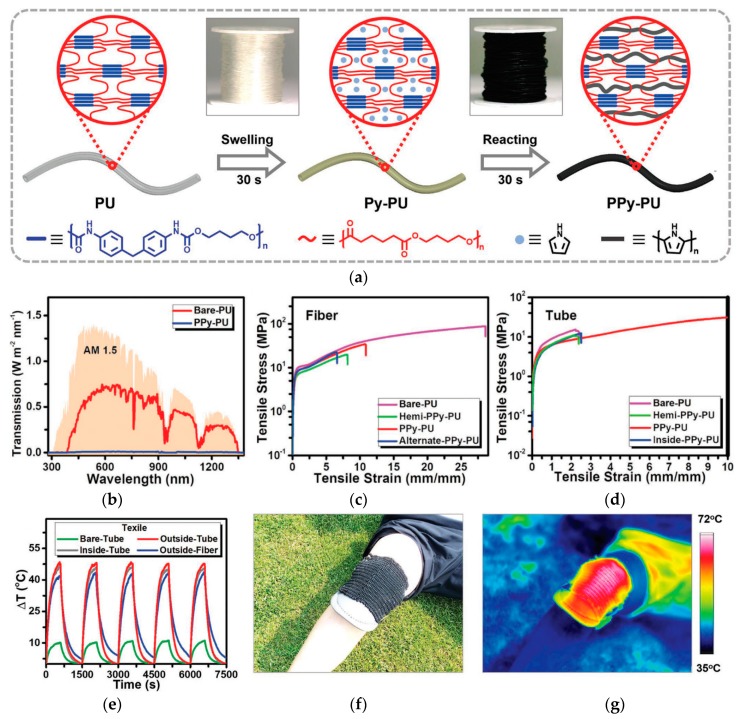
(**a**) Schematic illustration of the preparation process of PPy-PU elastomers, (**b**) transmittance power density of the bare-PU and the PPy-PU film, and the AM1.5 solar spectrum plotted as a reference. Tensile strain–stress curves of PU or PPy-PU, (**c**) fibers, (**d**) tubes, (**e**) temperature change of different textiles under cycles of on-off light illumination, (**f**) optical image of a photothermal kneecap for warming a knee joint in natural sunlight, and (**g**) the corresponding infrared image. Reproduced from Ref. [104] with permission. Copyright 2018, John Wiley and Sons.

The utilization of conjugated polymers for polymer nanocomposites, fibers, and textiles for photothermal purposes has been recently reported. Polypyrrole (PPy) is a representative conjugated polymer that can be synthesized via chemical oxidation of pyrrole monomers, typically using FeCl_3_ in a polar medium [103]. Wang et al. showed that the soaking of PU fibers or tubes in an aqueous solution of pyrrole monomers and FeCl_3_ oxidant could induce the swelling of soft segments in PU and the interpenetration of the monomers and oxidant into the PU elastomeric network structure (Figure 5) [104]. Confined polymerization of PPy in the PU network results in polymers with interpenetrating networks of PU and PPy. Textiles woven from the resulting PPy-PU fibers or tubes showed excellent photothermal properties because of efficient light absorption by PPy in the fibers or tubes under sunlight irradiation and the subsequent heat emission even at a low ambient temperature. Furthermore, because of the interpenetrated network structure at the molecular level, PPy-PU fibers and tubes presented an excellent tensile strain beyond 400%. 

**Figure 6:**
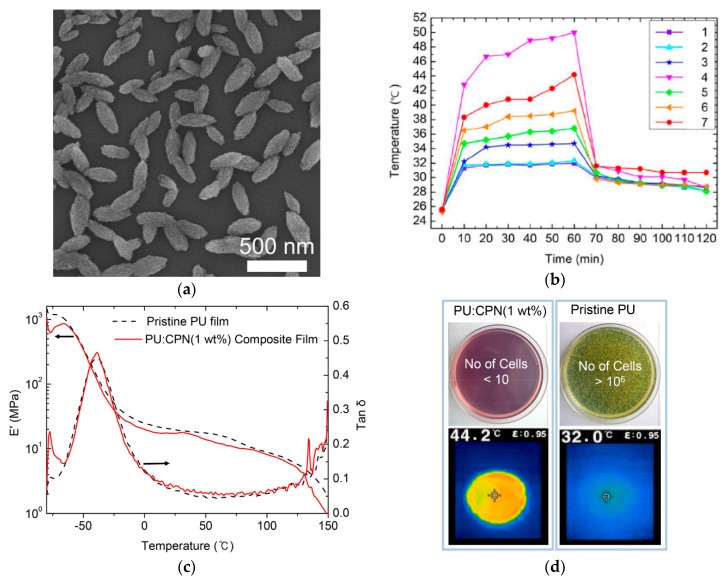
(**a**) Scanning electron microscopy (SEM) image of PCPDTBT nanoellipsoids, (**b**) temperature variation profiles under white light irradiation for 60 min and after turning off the light for another 60 min for (1) pristine PU, PU composite films with (2) 1 wt % OA, (3) 1 wt % Ag nanoparticles, (4) 1 wt % PCPDTBT, (5) 0.25 wt %, (6) 0.5 wt % and (7) 1 wt % CPNs, (**c**) dynamic mechanical analysis data of pristine PU and PU-CPN composite film at 1 wt %, and (**d**) optical images of bacterial cell cultures on films of PU-CPN composite and pristine PU films and infrared images after white light irradiation. Reproduced from Ref. [101] with permission. Copyright 2018, Elsevier.

Applications of a donor-accepter-type conjugated polymer for photothermal nanocomposites and fibers were presented using PCPDTBT [101]. To prepare CPNs, PCPDTBTs were first dissolved in chloroform, and the solution was added dropwise to a DMF solution containing octanoic acid (OA). The resulting emulsification of PCPDTBT with OA in the polar solvent, followed by removal of chloroform with heating at 80 °C, produced nanoellipsoids of PCPDTBT with average sizes of approximately 300 nm of the long axis and 120 nm of the short axis (Figure 6). PU-CPN nanocomposites were then prepared by mixing the dispersion of PCPDTBT nanoellipsoids in DMF and a PU DMF solution, followed by pouring the mixture solution on dishes and drying the solvent. At only 1 wt % CPN concentration, the PU-CPN nanocomposites showed a remarkable photothermal temperature increase of above 40 °C and a slight deterioration in the storage modulus of approximately 5 MPa in comparison to that of pristine PU film. Concurrently, the PU-CPN nanocomposite films showed antibacterial activity and reduced bacterial concentrations compared to silver nanoparticles due to the antimicrobial property of the OA as a fatty acid, thus presenting multifunctional nanocomposites. 

**Figure 7:**
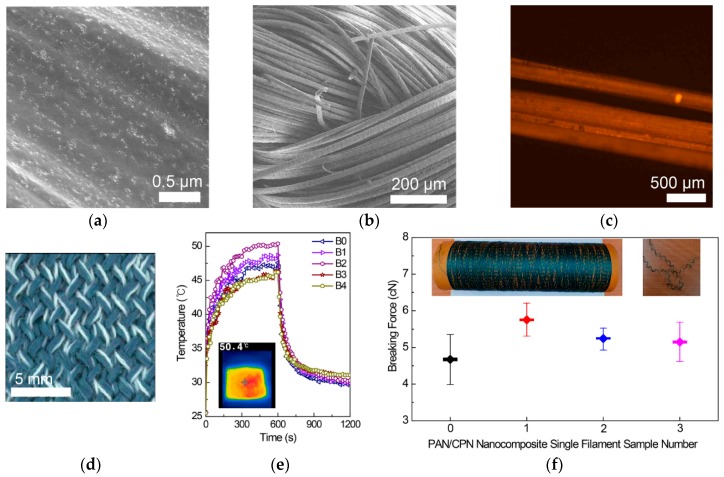
SEM images of (**a**) a nanocomposite fiber of PAN with 0.75 wt % CPNs, (**b**) fabric based nanocomposite fibers, (**c**) fluorescent (excited at 650 nm) images of yarn based on nanocomposite fibers, (**d**) an optical image of fabrics prepared from yarns based on PAN/CPN nanocomposite fibers, (**e**) photothermal response of the textiles fabricated using PAN/CPN nanocomposite fibers at 0.75 wt % of CPNs under white light irradiation for the first 600 s, followed by 600 s after turning off the light, with an infrared thermal image under white light irradiation at t = 600 s, and (**f**) breaking forces measured by single filament tensile experiments of pristine PAN fibers (filament 0), nanocomposite fibers before knitting the textiles (filament 1, left inset image), after knitting followed by deknitting without washing (filament 2), and after washing in a laundry machine followed by deknitting (filament 3, right inset image). Reproduced from Ref. [102].

Textiles based on CPNs have been presented using CPNs prepared from a solution spinning process. CPNs of PCPDTBT and OA have been prepared as a dispersion in DMSO via the emulsification process, and the resulting solution was mixed with a DMSO solution of polyacrylonitrile (PAN) at 18 wt % (Figure 7) [102]. When CPNs were formed via the emulsion process in DMSO, their shapes were spherical, with a diameter of 190 nm, which differed from the ellipsoidal shape after processing in DMF. This is because hydrogen bonds between DMF and carboxylic acids on the outermost surface of CPNs enhance the growth of the assembly structure in one direction. Spherical CPNs were distributed inside and on the outermost surfaces of PAN fibers due to favorable interactions between the PAN matrix and CPN fillers such as dipole-dipole interactions between cyano groups in the PAN and carboxylic groups in OA. Knitted textiles using yarns based on PAN-CPN nanocomposite fibers reached the highest temperature of 50.4 °C in 10 min of white light irradiation from a solar simulator. In the examination of antibacterial property, representative gram-positive and gram-negative bacteria, *Staphylococcus aureus* (*S. aureus*) and *Escherichia coli* (*E. coli*), were cultured on nanocomposite films containing only 0.7 wt % of CPNs. After 24 h, 99.9% of cells were eliminated, presenting the effect of OA, a fatty acid that enables the physical disruption of the cellular membrane structure, increases the fluidity and disorganization of the membrane, and thereby, disintegrates the bacterial cells. Importantly for practical use as a textile, breaking forces of PAN-CPN nanocomposite fibers were measured before and after washing in a laundry machine by single filament tensile experiments. The PAN-CPN fibers after machine washing presented a breaking force of 5.13 cN (165.94 MPa). This breaking force was higher than that of pristine PAN fibers (4.68 cN (151.28 MPa)) and within the range of the standard deviation for that of PAN-CPN fibers before washing (5.74 cN (185.78 MPa)), thereby showing the mechanical durability of the textiles during machine washing.

## 3. Functional Fibers and Textiles Based on Joule Heating

Polymer nanocomposites of conductive materials have been highlighted for the fabrication of stretchable, wearable electronic devices. Metallic nanoparticles and nanowires, carbon nanotubes (CNTs), graphene, and conducting polymers have been composited with elastomeric polymers such as styrene-butadiene-styrene (SBS) terpolymers, polydimethylsiloxane (PDMS), and PUs. Reviews regarding stretchable electronics and conductive nanocomposites are already available in the literature [105,106]. The Joule heating of fibers, textiles, and woven fabrics based on conductive materials has attracted increasing attention for warming and therapeutic applications. Wiring the fibers and textiles with conductive materials to external electrodes can generate Joule heating because of electrical currents and resistance. A voltage difference between two electrodes and the resulting electric field can generate a flow of charge carriers which are typically electrons that provide kinetic energy to the charge carriers. When charge carriers collide with ions in the conducting materials, the directional motion of the charge carriers is changed to thermal motion, converting the kinetic energy into thermal energy. As established by James P. Joule in the 1840s, the power of heating (*P*) is given as the product of a conductor’s resistance (*R*) and the square of the current (*I*, *P* = *I*^2^*R*) [107]. Metallic nanowires of silver (AgNW) and copper (CuNW), CNT, and reduced graphene oxides (RGOs) have been used as Joule heating materials for wearable heaters as films and in combination with fibers and textiles, which have only been recently pioneered. In this review, recent developments in composite films, fibers, and textiles based on Joule heating are described, categorizing them on the basis of methodology. 

### 3.1. Joule Heating Based on Films

**Figure 8:**
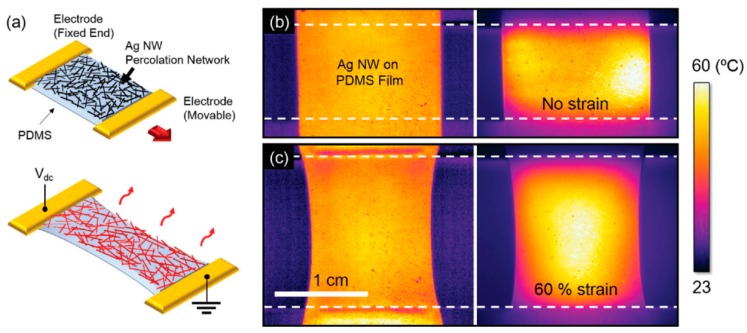
Highly stretchable and transparent heater. (**a**) Schematic illustration of the stretchable and transparent heater composed of an Ag NW percolation network on PDMS film, (**b**,**c**) pseudocolor image at room temperature (left) and infrared camera thermal image (right) of an Ag NW/PDMS stretchable and transparent heater operating at 60 °C with (**b**) no strain and (**c**) at 60% strain condition. Reproduced from Ref. [108] with permission. Copyright 2015, John Wiley and Sons.

**Figure 9:**
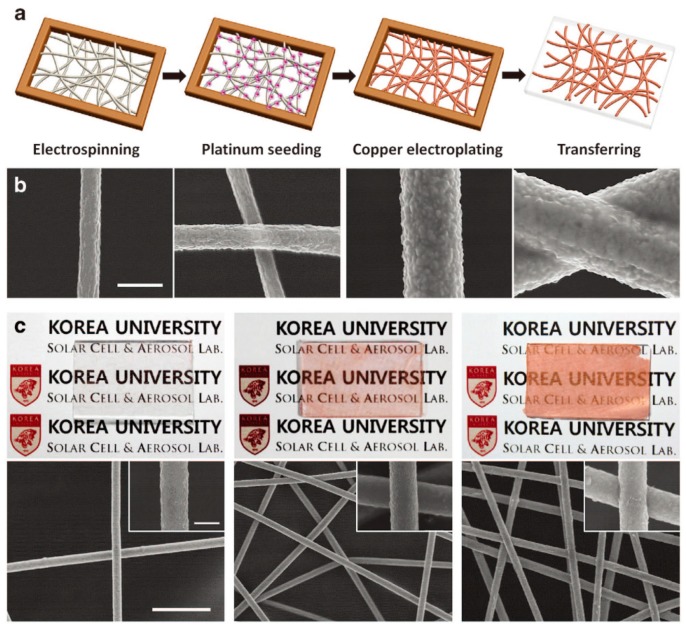
(**a**) Schematic representation of the CFH fabrication process. (**b**) SEM images of pristine PAN, platinum-seeded, Cu-electroplated, and transferred nanofibers. The scale bar is 1 μm, and (**c**) photographs and corresponding SEM images of the CFHs on glass substrates with first electrospinning times of 5, 90, and 180 s. The scale bars are 10 μm; those for the insets are 1 μm. Reproduced from Ref. [109].

Electrically driven Joule heaters have commonly been fabricated by forming percolated network structures of Ag NWs on polymer films or in polymer matrices. The resulting systems have initially been applied to planar substrates such as defogging/defrosting windows and later, to non-planar substrates such as wearable electronic devices and thermotherapy. In particular, highly flexible, stretchable, patternable, and transparent systems with efficient Joule heating properties are required for the latter applications. One of the most significant issues in the fabrication of composite films is forming the percolation structure on the polymer films or in the polymer matrix. A solution to this issue is to prepare percolated network structures of Ag NWs by vacuum filtering their ethanol solutions on polytetrafluoroethylene (PTFE) filters, followed by transferring the percolated structure onto a PDMS film upon contact of the Ag NW percolated film on the PTFT filter with a PDMS film (Figure 8) [108]. The resulting heater successfully operates under an elevated temperature of 60 °C and a strain of 60%. In a similar transfer technology, PAN nanofibers were first electrospun on a glass substrate followed by electroplating copper on the PAN nanofiber surfaces (Figure 9). An advantage of this process is that the electroplating process formed self-fused junctions in the percolated network structure of Cu NWs, significantly reducing contact resistance. The percolated structure of Cu-coated PAN nanofibers was transferred to a silicone-based Ecoflex rubber. The resulting heaters, based on Cu NWS, exhibited a temperature increase of up to 328 °C with 90% transparency and a sheet resistance of 0.058 Ωsq^−1^. Furthermore, they had a remarkable stretchability of up to 300% and durability after 1000 bending cycles without any deterioration in the Joule heating property. The Cu-plated nanofibers could be transferred onto any surface with a complex 3D structure. In contrast, a suspension of Ag nanoparticles and ethylene glycol was electrospun on plastic films of PI or PET, resulting in percolated Ag nanofibers upon annealing at a high temperature and under light irradiation [109]. 

**Figure 10:**
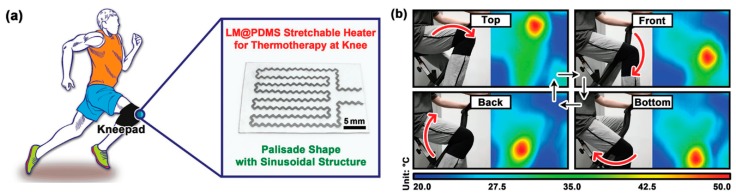
(**a**) Optical photo of a specially designed LM@PDMS stretchable wearable electronically driven heater (WEDH) and schematic illustration of its working condition. The pattern of this heater is palisade shape with sinusoidal structure to perform thermotherapy on the knee, and (**b**) optical photos of exercise at different states and the corresponding IR thermal images. Kneepad worn by a volunteer is embedded with the LM@PDMS stretchable WEDH of (**a**). Reproduced from Ref. [110] with permission. Copyright 2019, John Wiley and Sons.

**Figure 11:**
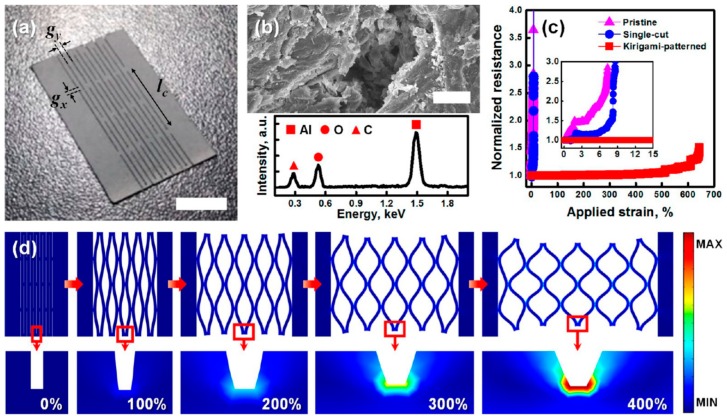
Kirigami-patterned stretchable paper conductor. (**a**) Digital image of the as-prepared stretchable Kirigami conductor (scale bar 10 mm), (**b**) SEM image and EDS spectrum characterized on the cut surface of the Al paper (scale bar 10 μm), (**c**) normalized electrical resistance of the pristine, single-cut, and Kirigami-patterned Al papers as a function of applied strain, and (**d**) FEM analysis of the stress distribution on the Kirigami conductor under various tensile strains. Reproduced from Ref. [111] with permission. Copyright 2017, American Chemical Society.

To enhance the stretchability and dynamic stability of a Joule heater, patterns of electrical conductors were incorporated into a polymer matrix. For example, a liquid metal (LM) alloy, based on gallium, indium, and tin (galinstan) was incorporated into PDMS films as sinusoidal patterns (Figure 10) [110]. A mixture of galinstan and PDMS was injected from a syringe onto a PDMS film to draw sinusoidal patterns. Liquid PDMS was then poured onto the PDMS film with LM patterns, followed by curing at a high temperature. Because of the highly conductive LM, its sinusoidal patterns, and 3D network in the PDMS matrix, the resulting composite film showed a high stretchability of over 100%, good conductivity of 1.81 × 10^3^ Scm^−1^, and excellent dynamic stability with only a slight change in electrical resistance and heating temperature of 4.23% and 7.56% upon 100% stretching. In a similar methodology, a kirigami pattern of a highly conductive Al paper was embedded in a highly elastic silicone elastomer (Ecoflex) (Figure 11) [111]. The pristine paper was immersed in an Al precursor solution, and the subsequent decomposition of the Al precursor led to Al coating on all fiber surfaces in the paper. After preparing the Al paper with the kirigami pattern simply by cutting the paper, the patterned paper was composited with Ecoflex. This simple approach enabled the resulting Joule heater to be extremely stretchable (>400% strain) and durable (1000 cycles at 300% strain) and to exhibit a high heating performance at low voltage (>40 °C at 1.2 V), thus demonstrating a Joule heater useful for a wearable thermotherapy device by increased blood flow at the wrist during operation. 

The next issue in the development of Joule heaters was to fabricate them via a rapid process. Blow spinning, laminating, and roll-to-roll processes have been combined with the percolated network structures of metal nanowires [112]. In a recent approach, Ag NWs were deposited onto a poly (ethylene terephthalate) (PET) substrate by a supersonic spray process. In contrast, PAN nanofibers were electrospun onto the other PET substrate, followed by electroplating Cu and nickel (Ni) onto the surface of the PAN nanofibers seeded with platinum (Pt) using a sputter. Two substrates with percolated network structures of different metal NWs were then laminated by placing them facing each other and superimposing, followed by roll-pressing and heating. The resulting conducting films exhibited a uniform distribution of Cu/Ni-plated PAN nanofibers and Ag NWs, a superior low sheet resistance of 0.18 Ωsq^−1^, a transparency of 91.1%, and controllable Joule heating performance. The film temperature could readily reach a high temperature beyond 300 °C by increasing the concentration of the nanofibers and NWs. In addition, aiming at applications for skin patches, thermotherapy, and temperature sensors, the film temperature could be controlled at a range below 50 °C by adjusting the applied voltages below 1.2 V and time to less than 10 s. In the blow spinning process, precursor solutions of Ag ions and poly(vinyl pyrrolidone) (PVP) were jetted from an eight-needle syringe module toward a rolling continuous polyimide (PI) track, followed by UV curing at room temperature [113]. The resulting Ag nanofibers, with a diameter of 650 nm presented a sheet resistance of 9.5 Ωsq^−1^ and a temperature increase of up to 285 °C at a direct current (DC) voltage of 10 V.

### 3.2. Joule Heating Based on the Coating of Textiles

**Figure 12:**
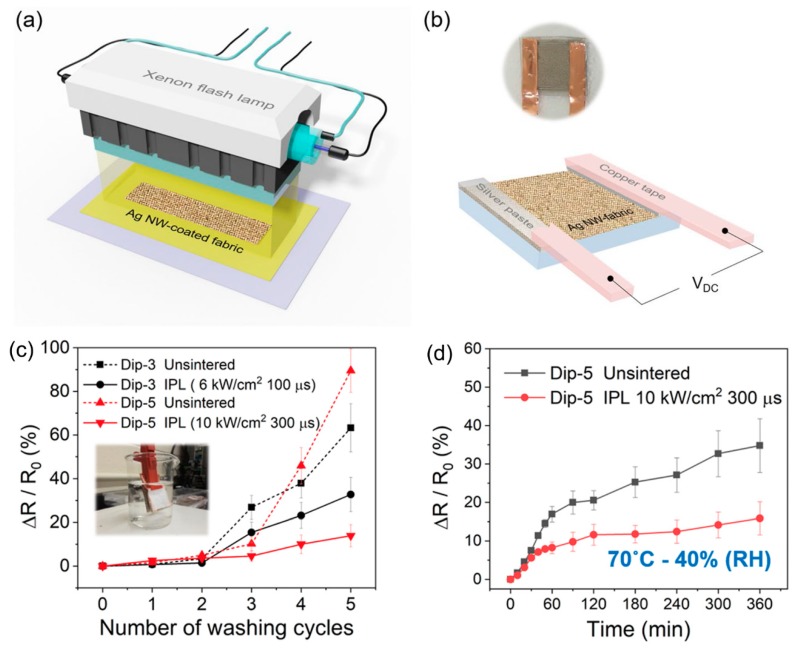
Schematic of (**a**) IPL sintering system for Ag nanowire-fabrics, (**b**) Ag nanowire-fabrics with copper tape adhered to the fabric using silver paste for applying DC voltage and measuring resistance of the fabric patch, (**c**) the normalized change in sheet resistance after a washing test for Dip-3 (black) and Dip-5 (red) Ag nanowire-fabrics before (dotted line) and after (solid line) IPL irradiation. Inset shows the washing test setup. The evolution of normalized change in sheet resistance for the Ag nanowire-fabrics before- (black) and after- (red) IPL irradiation under different temperature and humidity conditions, and (**d**) Dip-5, 70 °C-40% relative humidity, Dip-5 IPL parameters: 10 kW·cm^−2^, 300 μs. Reproduced from Ref. [115].

As another approach to fabricate Joule heaters, electrically conducting materials have been simply coated on textiles that are already manufactured using conventional fibers. In a previous study, Ag NWs were synthesized as a dispersion in ethanol solutions, and cleaned cotton fabrics were repeatedly immersed into the solutions until the sheet resistance reached 2.5 Ωsq^−1^, then dried and firmly attached with a silver paste and copper tape [114]. The temperature of the resulting textiles could be controlled between 30 and 125 °C, corresponding to the applied voltages between 1 and 6 V. The heating performance of the as-coated fabric remained preserved after 5000 bending cycles. However, it gradually decreased from 87 to 35 °C upon five repeated washing cycles because of the loss of Ag NWs coated on the fabric. The durability of the conducting fabrics could be improved by employing a post sintering process (Figure 12) [115]. Woven PET fabrics were cleaned and immersed into ethanol solutions of Ag NWs. After repeated dip coating of up to five cycles, the prepared Ag NW-coated fabrics were exposed to intense pulsed light (IPL) for sintering. After sintering, Ag NW-coated fabrics showed the lowest sheet resistance of 0.46 Ωsq^−1^, 30% less than that of unsintered fabrics. In addition, the increase in their electrical resistance (ΔR/R_0_) after five washing cycles was 16%, while that of unsintered fabrics was 84%. In the most recent study, an approach for quick processing was reported employing a spray process. A dispersion of Ag NWs and multi-walled CNTs was spray-coated onto a stretchable fabric substrate (80% nylon and 20% PU), followed by encapsulating with silicone rubber (Ecoflex) after drying [116]. The resulting fabrics presented a stretchability of 50%, a sheet resistance of 22 Ωsq^−1^, and operating temperatures of 35–55 °C at a low driving voltage of 3–5 V. As another type of conductive material for coating textiles, conductive nanoparticles were used to construct multifunctional wearable devices [117]. Cleaned cotton and wool fabrics were immersed in a dispersion of gold nanoparticles (GNPs) and carbon black (CB) with ultrasonication. The resulting coated fabrics were then encapsulated with Ecoflex, producing highly stretchable smart textiles with a strain of more than 100% and a maximum temperature elevation of up to 103 °C at 20 V. Carbon-based conductive inks, including CNTs [118,119] and graphene nanoplatelets [120], were coated directly on cotton or PET fabrics by a scalpel-printing method or a simple dip-and-dry process. The CNT-cotton fabric was used as a sensor for human motions such as walking, running, squatting and bending due to its large strain range (0~100%) and as an electric heater with a significant electric heating performance (78 °C at 20 V within 2 min) [118]. Woven textiles coated with a printable CNT concentrate was also examined for dye sensitized solar cells, together with Joule heating. They demonstrated 8% efficiency and a temperature increase of up to 120 °C at 20 V [119]. 

**Figure 13:**
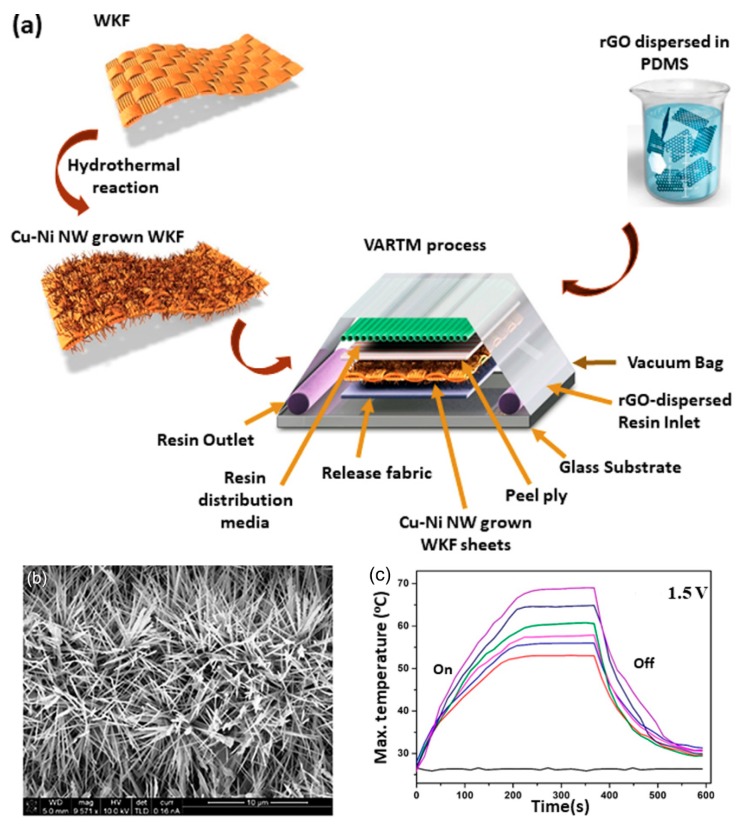
(**a**) Schematic diagram for preparation of the WKF/Cu−Ni/rGO/PDMS composite, (**b**) SEM image of Cu_3_Ni_1_ NWs, and (**c**) time-dependent temperature profile during Joule heating of WKF composites. Reproduced from Ref. [121] with permission. Copyright 2018, American Chemical Society.

In contrast, conductive materials can be coated on the surface of fabrics by chemical reactions. Vertical Cu-Ni NWs were directly grown on woven Kevlar fiber (WKF) via a hydrothermal reaction and combined with RGO dispersed in PDMS via a vacuum-assisted resin transfer molding (VARTM) process (Figure 13) [121]. The resulting composite exhibited excellent performance as a Joule heater, reaching 70 °C at 1.5 V. Furthermore, IR from the human body can be reflected back, thereby providing increased thermal insulation of 43%. Ni was also deposited onto silk fabrics [122]. Kapton tapes with a pre-cut interdigital pattern were attached to pre-cleaned silk fabrics, followed by an electroless plating process of Ni electrode patterns. A GO layer was spray-coated onto these Ni-patterned silk fabrics to act as a humidity sensor. The resulting fabrics could be applied as a flexible humidity sensor for monitoring human respiration. Transparent conducting films and heaters could be fabricated by coating PAN fibers prepared by electrospinning with Ni via an electroplating process [123]. The resulting mats of Ni-coated PAN fibers presented a remarkably low sheet resistance of 0.73 Ωsq^−1^ at an optical transmittance of 93%, achieving a heating temperature of 373 °C at an applied voltage of 2 V. As a representative organic conductive material, conjugated polymers such as polyaniline, poly(3,4-ethylenedioxythiophene) and PPy could be coated on fabrics by a vapor phase polymerization, an electrochemical polymerization, in-situ solution polymerization or inkjet/blade printing, as recently reviewed [124]. For example, PPy was synthesized on the surfaces of cotton and nylon fabrics via in-situ polymerization with FeCl_3_ [125,126,127]. The operating temperature of the resulting Joule heaters based on PPy-coated cotton fabrics was in the range of 28–83 °C at an operating voltage of 3–9 V with a sheet resistance of 303 Ωsq^−1^ [127] and demonstrated multifunctionalities with superhydrophobicity and self-cleaning effects [126]. 

### 3.3. Joule Heating Based on Conductive Fibers and Their Woven Fabrics

**Figure 14:**
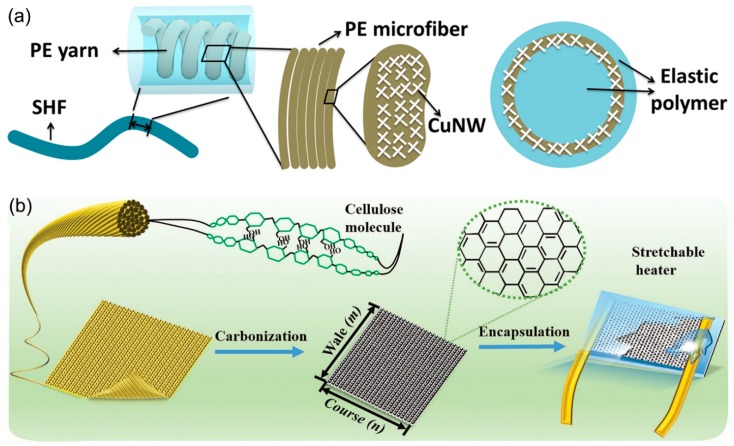
(**a**) Schematic illustration of the hierarchical structure of the SHF. Reproduced from Ref. [128] with permission. Copyright 2016, American Chemical Society, and (**b**) schematic illustration showing the fabrication process of the carbonized Modal textile heater. Reproduced from Ref. [129] with permission. Copyright 2017, John Wiley and Sons.

In addition to surface coating technologies by conductive materials, various technologies have been reported to prepare conductive fibers and use them as woven textiles. In a hierarchical structure of a stretchable heating fiber (SHF), Cu NWs were coated onto a helical yarn of polyester microfibers, followed by encapsulation of the fibers with silicone rubber (Figure 14a) [128]. The resulting SHF could reach a temperature range of 20–57 °C at a low applied voltage of 3 V, within 20 s. A Joule heater could be directly prepared from commercially available fabric (Figure 14b) [129]. Weft-knitted Modal fabric was carbonized under an argon and hydrogen mixed atmosphere at an elevated temperature of 1050 °C for 200 min, thus preserving the stretchability of the carbonized textile by slow heating and cooling. The carbonized fabrics were then wired with a Cu electrode and encapsulated by Ecoflex silicon rubber. The resulting stretchable heater showed a heating temperature higher than 100 °C at a driving voltage of as low as 3 V, without deterioration of the Joule heating performance, even under a large strain of 70%. 

## 4. Thermal Management Based on Biomimetic Control over Fabric Structures

**Figure 15:**
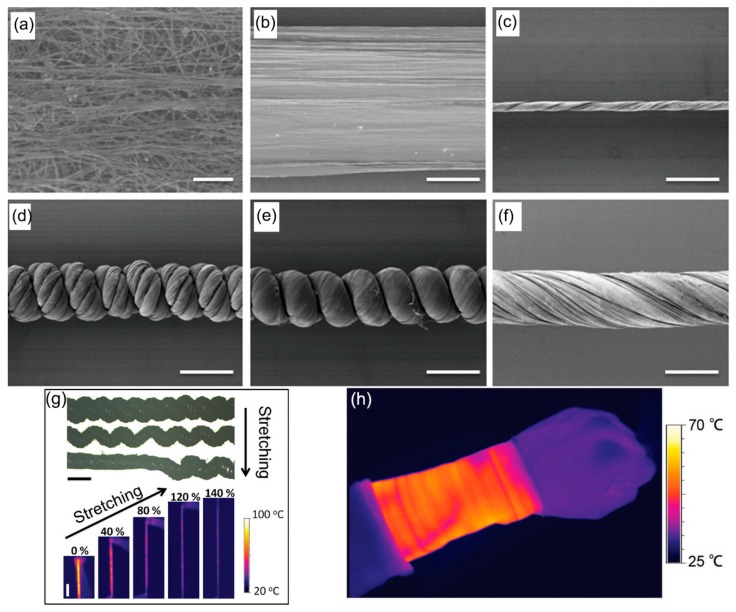
Scanning electron microscope (SEM) images of CNT ribbons at (**a**) high and (**b**) low magnifications, respectively, (**c**) SEM images of primary CNT fiber, (**d**–**f**) SEM images of HHF, single-ply spring-like fiber, and single-ply nonspring-like fiber with the same diameter. Scale bars: 1 μm in (**a**) and 200 μm in (**b**−**f**). (**g**) Optical images and infrared images during stretching. Scale bars: 200 μm (top) and 1 cm (bottom). (**h**) Infrared image of the heating textile wrapped on the wrist at 9 V. Scale bar: 2.5 cm. Reproduced from Ref. [130] with permission. Copyright 2017, John Wiley and Sons.

Smart fibers that mimic the structures in nature have most recently been highlighted for enhancing thermal storage and absorption in combinations with Joule heaters or natural heat sources such as the human body. A hierarchical structure that mimics the helical structure of the classic thermal insulation material, wool, was developed using CNT (Figure 15) [130]. Ribbons of CNT were first synthesized, dried, twisted, and collected onto a spool to produce CNT fibers. A bundle of the CNT fibers was then twisted to fabricate compact helixes. The resulting textiles woven from the hierarchically helical fibers (HHFs) of CNT could be stretched up to 150% with high stability and reversibility and could exhibit ultrafast thermal response over 1000 °C s^−1^ at a low operating voltage of several volts and heating durability over 5000 cycles. Furthermore, they had a good thermal insulation property due to a large number of voids in the hierarchically helical structure. 

**Figure 16:**
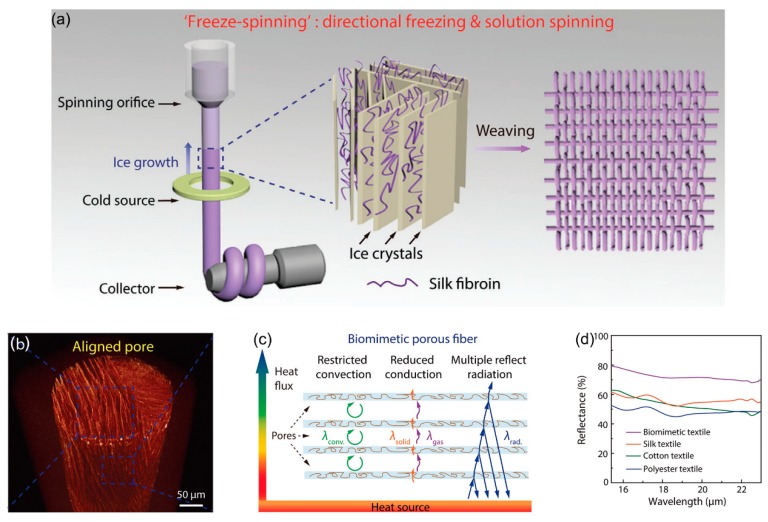
(**a**) Schematic illustration of the “freeze spinning” technique, combining “directional freezing” with “solution spinning” to realize continuous and large-scale fabrication of biomimetic fibers with aligned porous structure, mimicking polar bear hair. The silk fibroin solution extruded from a pump-controlled syringe is gradually frozen when it passes through a cold copper ring. Collected by a motor, the frozen fiber is further freeze-dried to reserve its porous structure and subsequently woven into a textile, (**b**) X-ray computed microtomography image showing the aligned lamellar pores within the biomimetic fiber along its axial direction, (**c**) schematic illustration of thermal conductivity of the biomimetic porous fiber with aligned pores, and (**d**) infrared light reflectance measurement of different textiles with similar thicknesses (≈ 0.4 mm) performed using a Fourier transform infrared (FTIR) spectroscopy microscope: biomimetic, silk, cotton, and polyester textiles are compared. Reproduced from Ref. [131] with permission. Copyright 2018, John Wiley and Sons.

A similar approach for improving the thermal insulating properties by introducing voids in fibers was inspired by animals such as polar bears living in extremely cold environments (Figure 16) [131]. A porous fiber structure mimicking polar bear hair with a hollow core and aligned shell was constructed by combining directional freezing with solution spinning. An aqueous-based mixture solution of silk fibroin and chitosan extruded from a syringe was gradually frozen, followed by freeze-drying. As a result, ice crystals that were formed along the axial direction of spinning upon freezing were selectively removed to preserve the porous structure. The textiles subsequently woven and doped with CNTs presented a fast thermal response and uniform Joule heating, together with an excellent thermal insulation property for comfortable wear. When the textiles are brought into contact with the human body, four different heat transfer mechanisms are possible, including conduction and convection through the air in the hollow micropore structures, conduction across the solid fibers, and radiation (reflection) at the surfaces of the fibers coated with CNTs. Due to the air in the micropores, with 87% porosity being blocked by the fiber walls, the convective heat transfer in the pore becomes negligible. However, the thermal conductivity of air is generally much less than that of solids, thereby significantly decreasing the overall heat transfer. Furthermore, the CNT coating on the textiles enhances the reflection of radiation from the human body, and 20% more radiation is reflected than that by pristine textiles based on silk, cotton, and PET. As an overall result, the thermal insulation property of the biomimetic textiles was greatly enhanced, presenting a temperature difference between textile surface and the heating stage of as high as 14 and 20 °C when the stage temperature was based from room temperature to –20 to 80 °C, respectively.

## 5. Conclusions and Outlook

Films, fibers, and textiles, combined with photothermal and electrically conductive materials, have attracted significant attention because of their unique functionalities of heat generation for human clothing. This review described the recent innovations in this issue, with a focus on reports in the last three years and was categorized into three subjects; heat generation technologies of a passive type using light irradiation of the outside environment (photothermal heating), and those of an active type employing external electrical circuits (Joule heating). Photothermal approaches use ceramics, semiconducting polymers, and metal oxide nanomaterials that can efficiently absorb a vast amount of solar energy arriving at the earth’s surface from the Sun. These materials also efficiently emit their absorbed energy as heat rather than light, exhibiting an efficient photothermal conversion. One of the advantages of these technologies is that the materials can be readily incorporated into conventional polymeric fibers as fillers, while preserving the mechanical performance of pristine fibers. Thus, it is possible to apply these materials for commercialization. In contrast, the resulting fabrics generate heat only when they are exposed to sunlight. They can therefore be applied to outdoor clothes, goods, or military uses in harshly cold environments with abundant sunlight, and thermotherapy using a medical IR lamp. Fabrics utilizing Joule heating materials can actively produce heat by applying only a small voltage, and the temperature increase can be sensitively controlled up to a few hundred degrees Celsius. Hence, they are useful for defreezing or defogging applications as composite films, heating patches for thermotherapy, and wearable heating devices. Joule heating materials have typically been coated on fiber or textile surfaces because of the difficulty in preserving the electrical conductance when they are composited within conventional fibers as fillers. Therefore, novel technologies that can combine Joule heating materials with conventional fiber production processes are required for commercialization. 

In future studies, heat generation technologies should be combined with other kinds of thermal management methodologies and consider the three mechanisms of conduction, convection and radiation. As recently reviewed for the enhanced thermoregulating performance of fibrous materials, heat transfer processes occur in a route from human skin surface, air gap, cloth and environment. In the air gap between the human skin and inside surface of the cloth, conductive heat transfer is a major mechanism [15,131]. Furthermore, heat radiation from the human skin with a wavelength at approximately 9.5 μm and reflection of the radiation on the inside surface of the cloth can be controlled in the air gap [132]. Through the cloth to the environment, radiation from the human body, air and moisture transport, conductive and convective heat transfer, and active heat generation based on the photothermal or Joule heating, clothing should be controlled for overall personal thermal management. The biomimetic structures incorporated into textiles to increase voids suggest a good example for future study. Fabrication processes for fibers and textiles with porous inner structures and reflective surface structures should be combined with photothermal and Joule heating materials to maximize heat generation and to minimize heat loss across fabrics. This requires an understanding of the conduction, convection, and radiation of heat when designing the structures. Conversely, composite films or hydrogels bearing photothermal or Joule heating materials should be developed for transdermal drug delivery and cosmetic applications. Heating on wound spots also increases bacterial growth, although wound healing effects become significant due to the enhanced blood flow, and therefore, functions to inhibit the bacterial growth or to clean the bacterial cells but boost the growth of epidermal cells for healing of the wound need to be incorporated.
